# First case of periprosthetic joint infection due to *Clostridioides difficile* in China

**DOI:** 10.1186/s12879-021-06171-y

**Published:** 2021-05-21

**Authors:** Yang Song, Hong Yi Shao, Xiang Cheng, Yu Guo

**Affiliations:** 1 Department of Orthopaedic, Jishuitan Hospital and Fourth Medical College of Peking University, Beijing, China; 2 Department of Microbiology and Molecule Laboratory, Jishuitan Hospital and Fourth Medical College of Peking University, 31 East Street, Xinjiekou, Xicheng District, Beijing, 100035 CN China

**Keywords:** Periprosthetic joint infection, *Clostridioides difficile*, Antibiotic treatment, Case report

## Abstract

**Background:**

*Clostridioides difficile* usually causes intestinal infections. However, a 75-year-old lady had a periprosthetic joint infection due to this microorganism. We report a *C. difficile* infection of a prosthetic hip joint. Such an infection is rarely reported around the world.

**Case presentation:**

The elder female patient presented with a 2-year history of right hip pain with movement restriction. Her right leg was shorter than another. The skin around the right hip joint was red and swollen without sinus. Her lab test result showed elevator ESR and CRP. Her X-ray film showed a massive bone defect. The patient had a total hip arthroplasty 16years ago and had a revision 5 years ago. During this hospitalization, her cultures of the synovial fluid and tissue repeatedly grew *C. difficile*. She improved following two-stage revision surgery and antibiotic treatment. The patient has no recurrence of infection after a one-year follow-up.

**Conclusion:**

A rapid and accurate sample collection is significant for culture results, making an outstanding contribution to the successful treatment.

## Background

Total hip arthroplasty (THA) is a very effective surgery for the treatment of late-stage osteoarthritis. However, periprosthetic joint infection (PJI) is a potentially catastrophic complication that affects nearly 1 to 3% of patients who have undergone THA [1, 2]. Gram-positive cocci constitute more than two-thirds of the pathogenic organisms in PJI; other cases involve Gram-negative bacteria, fungi, or other organisms [3]. There are 35% [4, 5] of cases of prosthetic joint infection without detection of the infectious agent. *Clostridioides difficile* is a spore-forming, toxin-producing Gram-positive anaerobe, which usually causes intestinal infections, has rarely been reported in cases of PJI. When anaerobic culture is applied, *C. difficile* is well culturable. Only five cases [6,10] of PJI involving *C. difficile* have been reported in the literature to date. Among them, two were ultimately resolved by amputation. To the best of our knowledge, no such cases have been reported in China. We herein report the first known case of PJI caused by *C. difficile* after THA in China and describe the isolation technique and treatment of this microorganism.

## Case presentation

A 75-year-old woman presented with a 2-year history of right hip pain with restriction of movement. She had undergone right hip arthroplasty because of a traumatic fracture 16years previously;5years ago, she underwent a revision for joint pain and limit movement. She had no comorbidities. Her hip pain had recurred 2years ago, and she was treated with second-generation cephalosporin for her symptoms (night pain, elevated temperature, swollen area of the hip), and her erythrocyte sedimentation rate (ESR) was 60mm/h (normal range 0~10mm/h), and her C-reactive protein (CRP) concentration was 304mg/L (normal range 0~10mg/L). The lab test result came to normal, but the pain still existed after the antibiotic treatment. Her pain and disability had increased during the most recent 2months.

**Fig. 1:**
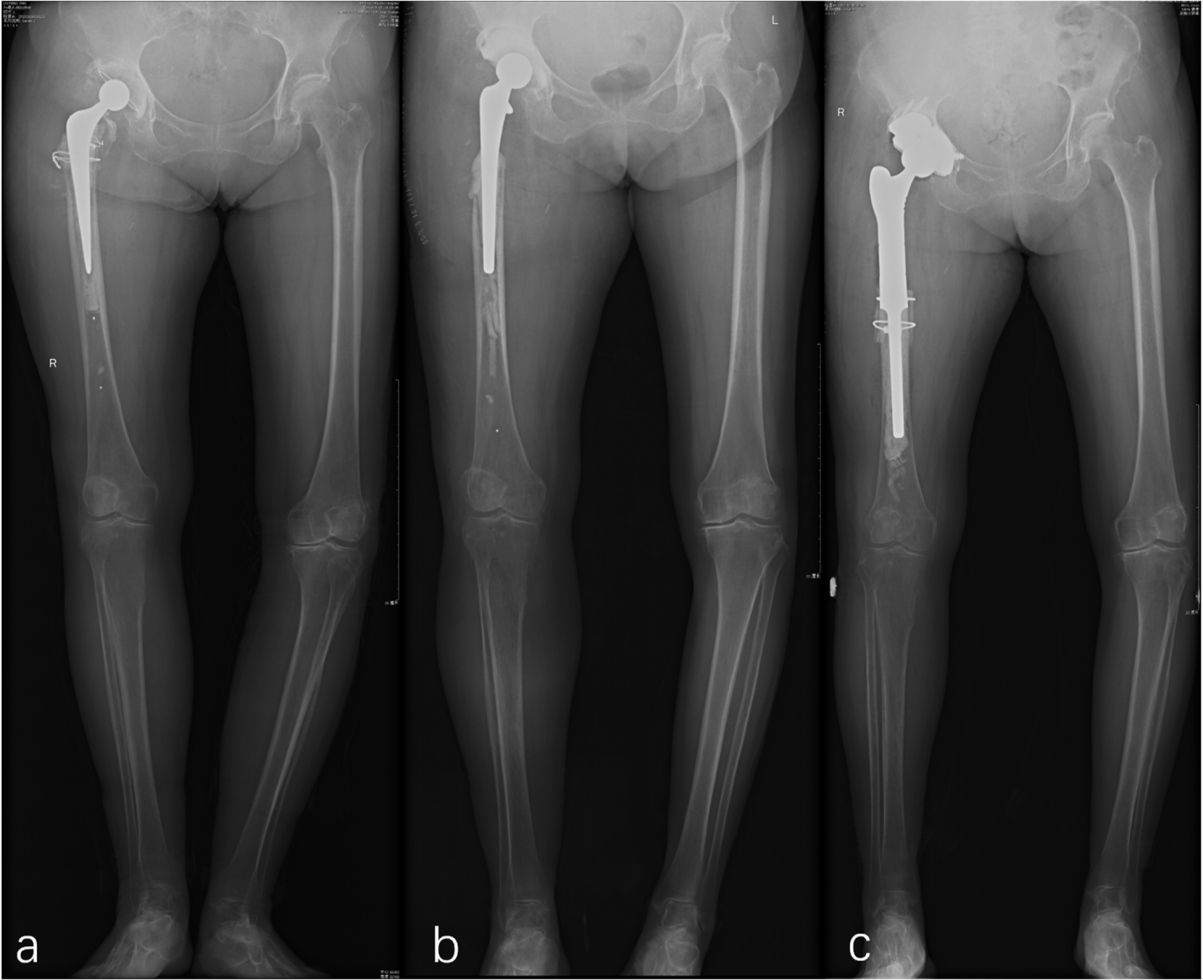
X-ray films of this two-stage treatment. **a**: X-ray film of the patient before the first stage operation; **b**: X-ray film of the patient after the first stage operation; **c**: X-ray film of the patient before the second stage operation

The patient arrived at our hospital in a wheelchair. Physical examination showed that her right lower extremity was shortened and that her hip exhibited flexion contracture; the skin around the hip joint was red, swollen without sinus. Her preoperative ESR was 43mm/h, and her CRP was 20.4mg/L. Before the first stage, the X-ray film showed a massive bone defect of the proximal femur, making the leg shortened (Fig. 1a).

**Fig. 2:**
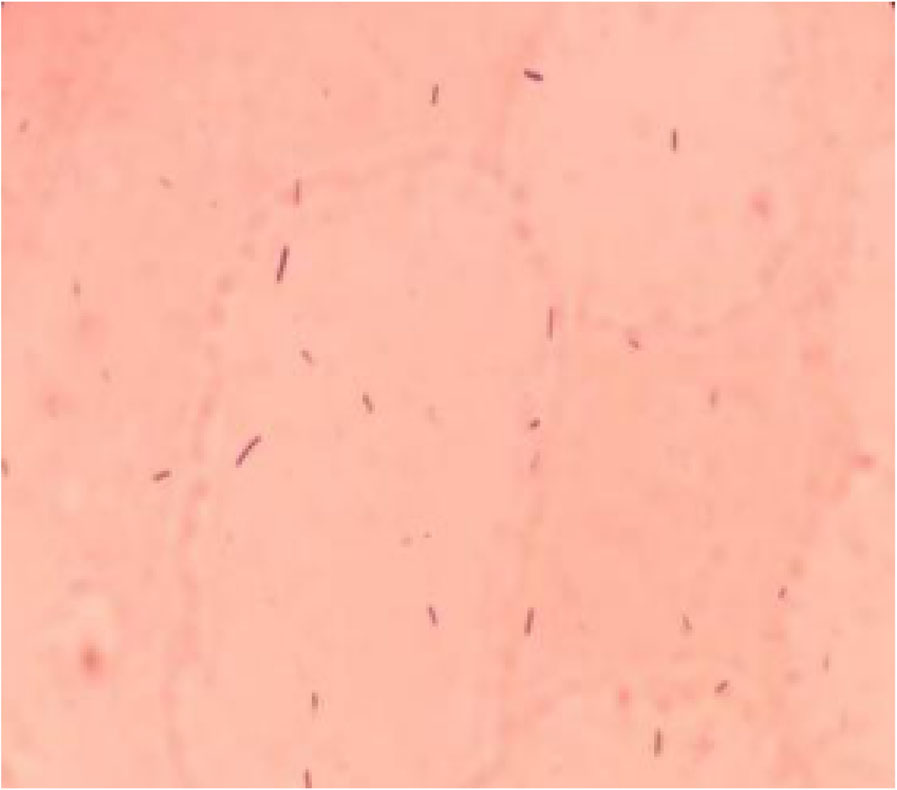
Gram-stain, microscopic morphology of *Clostridium difficile* (1000)

**Fig. 3:**
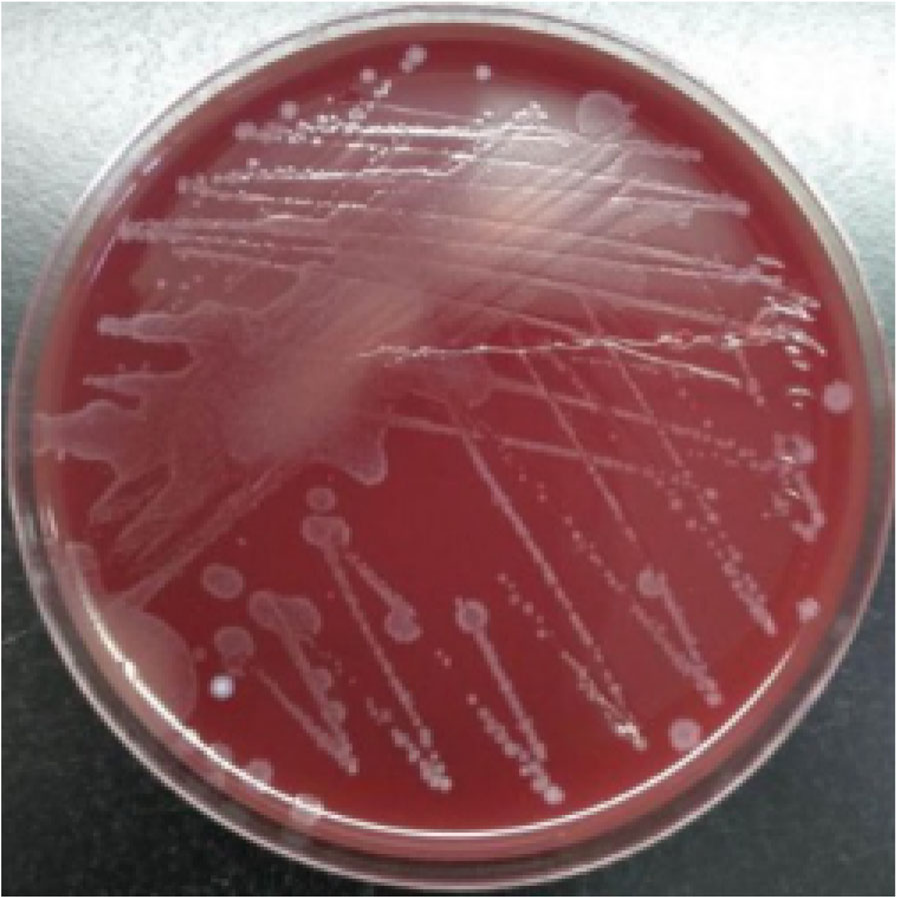
Colony morphology of *Clostridium difficile* on anaerobic blood agar (24-h culture)

Aspiration of the patients hip joint produced almost 30mL of synovial fluid. The synovial fluid examination revealed a white cell count (WBC) of 7548/mm3 with a polymorphonuclear neutrophil (PMN) percentage of 77%. We inoculated samples of the synovial fluid into four blood culture vials (two aerobic and two anaerobic vials) and cultured them using an instrumented blood culture system (BACTECTM FX 100, Becton, Dickinson and Company, New Jersey, US). The two anaerobic culture vials exhibited growth at 44 and 62h after inoculation, respectively; we then performed a Gram stain of the samples and found Gram-positive bacilli under microscopy (Fig.2). Gray-white wet colonies of medium size, round shape, and irregular edges were observed in the anaerobic medium after 24h of incubation (Fig.3). The organism was identified as *C. difficile* by matrix-assisted laser desorption/ionization time-of-flight mass spectrometry (MALDI-TOF MS, Bruker Corporation, Nehren, Germany). Identification was confirmed by 16S rRNA sequencing. Polymerase chain reaction tested (Tsingke company, Beijing, China) positively for the *tcdA* and *tcdB* genes of *C. difficile*. Antimicrobial testing showed that the bacterium was sensitive to metronidazole and vancomycin (Etest, BIOMERIEUX, Paris, France).

We performed a two-stage revision for treatment of this patient. In the first stage, we removed the prosthesis and performed thorough debridement; this was followed by the placement of a cement spacer mixed with vancomycin (Fig.1b). We mixed 4g vancomycin in 36g cement (PALACOSR, Heraeus Medical GmbH, Wehrheim, Germany). Intraoperative cultures of the synovial fluid and tissue repeatedly grew *C. difficile*. The antimicrobial susceptibility results were unchanged from the previous cultures. According to the results of antimicrobial testing, metronidazole was selected for treatment. The patient was treated with intravenous metronidazole for 2weeks postoperatively and oral metronidazole (400mg three times a day) for another 4weeks. She then underwent the second stage of hip reconstruction after confirming that her laboratory parameters were normal (CRP: 2.65mg/L; ESR: 26mm/h). We used a tumor prosthesis to reconstruct her femur bone defect. And we used augment to econstruct the acetabular (Fig.1c). We obtained intraoperative samples again to ensure that the infection was under control. We checked an intraoperative cell count (WBC: 247/mm^3^; PMN percentage: 7%). Cultures of all samples showed no growth, and the patient underwent another round of antibiotic treatment (2weeks of intravenous metronidazole and another 4weeks of oral metronidazole, as before). She returned for regular follow-ups. At her latest follow-up, 1year after the diagnosis of PJI due to *C. difficile*, her right hip was pain-free, and the incision had healed without clinical signs of infection.

## Discussion and conclusions

*Clostridioides difficile* (previously termed *Clostridium difficile*) is a pathogen that causes antibiotic-associated diarrhea and pseudomembranous colitis with clinical manifestations of diarrhea, abdominal pain [11]. Significant risk factors for developing *C. difficile* infection (CDI) include antibiotic use, older age, poor host immune function, proton pump inhibitor use, previous CDI, and diabetes [12]. However, few extraintestinal CDI cases have been reported, especially bone and joint infections such as septic arthritis, osteomyelitis, and PJI. To date, only five patients have been reported to have PJI due to *C. difficile*; among them, two ultimately required amputation [6,10]. One of these cases was reported in 1995; a 16-year-old boy developed CDI after total knee arthroplasty in France and underwent leg amputation 1year later. The other case involved a 61-year-old woman in the United States; a strain of *C. difficile* was isolated from tissue and fluid aspirated from her knee. The patient underwent an above-knee amputation 3months later. In such cases, the reason for treatment failure is speculated to be associated with bacterial virulence and high antibiotic resistance. We have summarized all previously reported cases in Table1.

**Table 1:** Summary of Cases

	Case 1 [6]	Case 2 [7]	Case 3 [8]	Case 4 [9]	Case 5 [10]	This case
Region/Time	France/1995	Australia/1999	America/2013	America/2013	Belgium/2014	China/2019
Patients age and sex	16-year-old man	83-year-old woman	61-year-old woman	47-year-old woman	61-year-old man	75-year-old woman
Site of infection	knee	hip	knee	shoulder	hip	hip
Type of infection	late (16months later)	delayed (12months later)	early (3months later)	early (3months later)	early (1week later)	late (24months later)
Surgical treatment	arthrotomy with drainage, removal of implant	Revision of the hip and removal of implant	Removal of implant	Debridement with removal of the hardware	Debridement, retention	Debridement, two-stage exchange arthroplasty
Antibiotic treatment	Amoxicillin/ornidazole/rigampicin/lincomycin/penicillin G	Metronidazole	Piperacillin-tazobactam/Metronidazole	Vancomycin/Metronidazole	Vancomycin/Metronidazole	Vancomycin/Metronidazole
Outcome	Amputation	Successful	Amputation	Unknown	Successful	Successful

Our patient had undergone long-term administration of various antibiotics, which substantially increased her risk of CDI. Because we isolated the same strain of *C. difficile* from both the preoperative synovial fluid and intraoperative tissue culture, we were sure that the *C. difficile* was the pathogenic organism of the PJI and was not a contaminant. However, the patient developed no diarrhea or other gastrointestinal symptoms during her hospitalization, and we did not examine her feces. Because there was no directly anatomical way between the abdominal and the hip joint, we speculate that the *C. difficile* had reached the hip joint by hematogenous spread as previously described [13].

We considered that the culture result would be the critical factor in a successful outcome during our patients treatment. Previous reports have shown that the most critical risk factor for misdiagnosis of CDI is the process of sample collection [14]. If this collection process is not done quickly and correctly, the test results may be strongly influenced. In particular, because *C. difficile* is a Gram-positive anaerobe, it should be kept in an anaerobic environment. Previous research has shown that testing such samples should be completed in 2h to increase the positivity rate [15]. Blood culture vials are commonly used to store synovial fluid samples in the hospital [16]; however, storage duration before testing in the clinical laboratory may be too long. There was a special recommendation [17] for treating CDI in intestinal but the joint. Metronidazole and vancomycin are recommended for the treatment of colonic CDI by the Infectious Diseases Society of America [18]. Our patient underwent two-stage exchange arthroplasty combined with a long antibiotic therapy course and showed a good outcome after 1year of follow-up.

In conclusion, rapid sample collection is critically important in the clinical setting. An appropriate surgical technique and metronidazole anti-infection therapy significantly contributed to the successful result in this case.

## Data Availability

Data sharing is not applicable to this article as no datasets were generated or analyzed as a part of this case report.

## Abbreviations

THA: Total hip arthroplasty
PJI: Periprosthetic joint infection
*C. difficile*: *Clostridioides difficile*
CDI: *Clostridioides difficile* infection
CRP: C-reactive protein
ESR: Erythrocyte sedimentation rate
WBC: White cell count
PMN: Polymorphonuclear neutrophil
